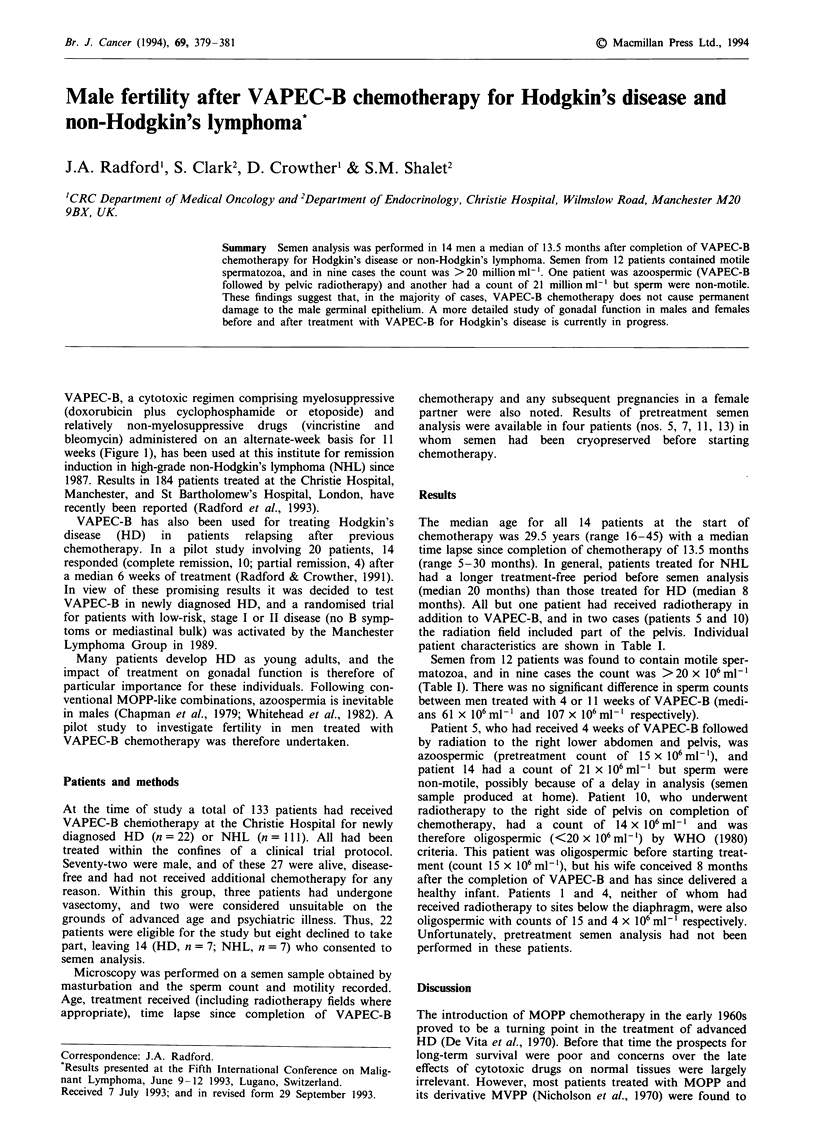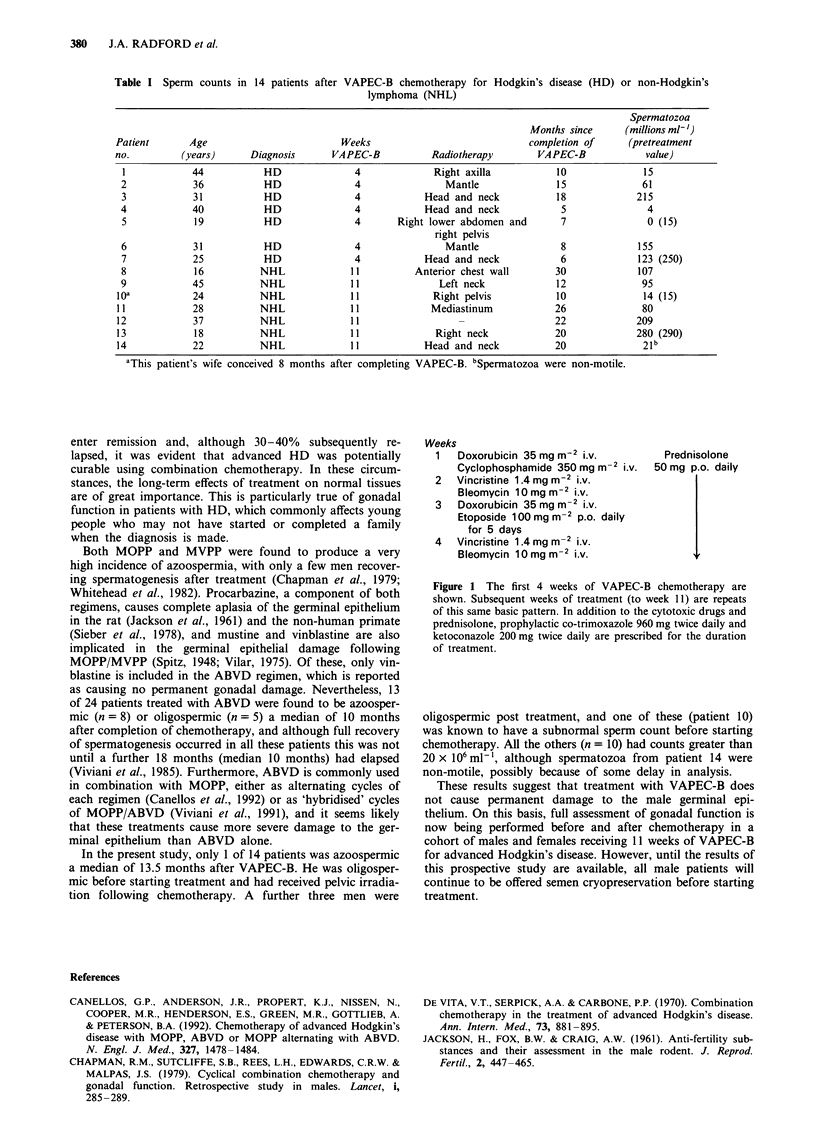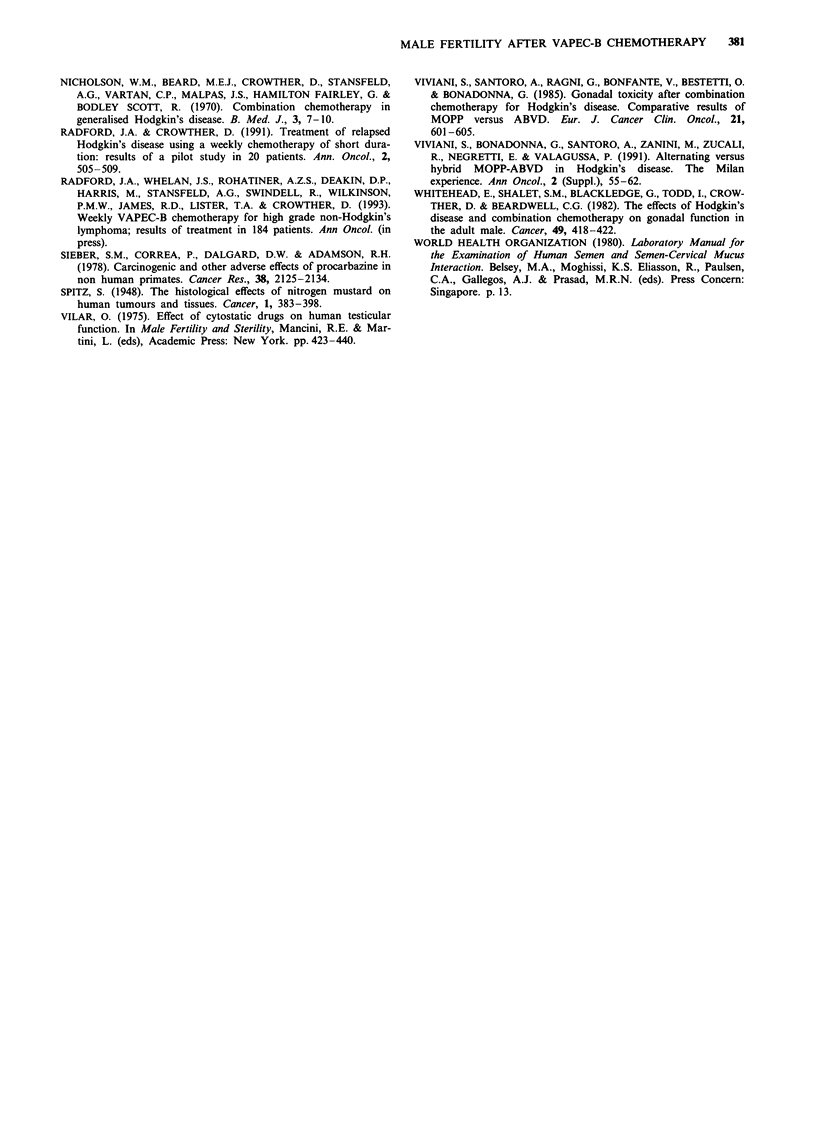# Male fertility after VAPEC-B chemotherapy for Hodgkin's disease and non-Hodgkin's lymphoma.

**DOI:** 10.1038/bjc.1994.69

**Published:** 1994-02

**Authors:** J. A. Radford, S. Clark, D. Crowther, S. M. Shalet

**Affiliations:** CRC Department of Medical Oncology, Christie Hospital, Manchester, UK.

## Abstract

Semen analysis was performed in 14 men a median of 13.5 months after completion of VAPEC-B chemotherapy for Hodgkin's disease or non-Hodgkin's lymphoma. Semen from 12 patients contained motile spermatozoa, and in nine cases the count was > 20 million ml-1. One patient was azoospermic (VAPEC-B followed by pelvic radiotherapy) and another had a count of 21 million ml-1 but sperm were non-motile. These findings suggest that, in the majority of cases, VAPEC-B chemotherapy does not cause permanent damage to the male germinal epithelium. A more detailed study of gonadal function in males and females before and after treatment with VAPEC-B for Hodgkin's disease is currently in progress.


Br. J. Cancer (1994), 69, 379–381 © Macmillan Press Ltd., 1994

### Male fertility after VAPEC-B chemotherapy for Hodgkin's disease and non-Hodgkin's lymphoma*

J.A. Radford[1], S. Clark[2], D. Crowther[1] & S.M. Shalet[2]

[1]CRC Department of Medical Oncology and [2]Department of Endocrinology, Christie Hospital, Wilmslow Road, Manchester M20 9BX, UK.

**Summary** Semen analysis was performed in 14 men a median of 13.5 months after completion of VAPEC-B chemotherapy for Hodgkin's disease or non-Hodgkin's lymphoma. Semen from 12 patients contained motile spermatozoa, and in nine cases the count was >20 million $ml^{-1}$. One patient was azoospermic (VAPEC-B followed by pelvic radiotherapy) and another had a count of 21 million $ml^{-1}$ but sperm were non-motile. These findings suggest that, in the majority of cases, VAPEC-B chemotherapy does not cause permanent damage to the male germinal epithelium. A more detailed study of gonadal function in males and females before and after treatment with VAPEC-B for Hodgkin's disease is currently in progress.

VAPEC-B, a cytotoxic regimen comprising myelosuppressive (doxorubicin plus cyclophosphamide or etoposide) and relatively non-myelosuppressive drugs (vincristine and bleomycin) administered on an alternate-week basis for 11 weeks (Figure 1), has been used at this institute for remission induction in high-grade non-Hodgkin's lymphoma (NHL) since 1987. Results in 184 patients treated at the Christie Hospital, Manchester, and St Bartholomew's Hospital, London, have recently been reported (Radford *et al.*, 1993).

VAPEC-B has also been used for treating Hodgkin's disease (HD) in patients relapsing after previous chemotherapy. In a pilot study involving 20 patients, 14 responded (complete remission, 10; partial remission, 4) after a median 6 weeks of treatment (Radford & Crowther, 1991). In view of these promising results it was decided to test VAPEC-B in newly diagnosed HD, and a randomised trial for patients with low-risk, stage I or II disease (no B symptoms or mediastinal bulk) was activated by the Manchester Lymphoma Group in 1989.

Many patients develop HD as young adults, and the impact of treatment on gonadal function is therefore of particular importance for these individuals. Following conventional MOPP-like combinations, azoospermia is inevitable in males (Chapman *et al.*, 1979; Whitehead *et al.*, 1982). A pilot study to investigate fertility in men treated with VAPEC-B chemotherapy was therefore undertaken.

### Patients and methods

At the time of study a total of 133 patients had received VAPEC-B chemotherapy at the Christie Hospital for newly diagnosed HD ($n = 22$) or NHL ($n = 111$). All had been treated within the confines of a clinical trial protocol. Seventy-two were male, and of these 27 were alive, disease-free and had not received additional chemotherapy for any reason. Within this group, three patients had undergone vasectomy, and two were considered unsuitable on the grounds of advanced age and psychiatric illness. Thus, 22 patients were eligible for the study but eight declined to take part, leaving 14 (HD, $n = 7$; NHL, $n = 7$) who consented to semen analysis.

Microscopy was performed on a semen sample obtained by masturbation and the sperm count and motility recorded. Age, treatment received (including radiotherapy fields where appropriate), time lapse since completion of VAPEC-B chemotherapy and any subsequent pregnancies in a female partner were also noted. Results of pretreatment semen analysis were available in four patients (nos. 5, 7, 11, 13) in whom semen had been cryopreserved before starting chemotherapy.

### Results

The median age for all 14 patients at the start of chemotherapy was 29.5 years (range 16–45) with a median time lapse since completion of chemotherapy of 13.5 months (range 5–30 months). In general, patients treated for NHL had a longer treatment-free period before semen analysis (median 20 months) than those treated for HD (median 8 months). All but one patient had received radiotherapy in addition to VAPEC-B, and in two cases (patients 5 and 10) the radiation field included part of the pelvis. Individual patient characteristics are shown in Table I.

Semen from 12 patients was found to contain motile spermatozoa, and in nine cases the count was $>20 \times 10^6 ml^{-1}$ (Table I). There was no significant difference in sperm counts between men treated with 4 or 11 weeks of VAPEC-B (medians $61 \times 10^6 ml^{-1}$ and $107 \times 10^6 ml^{-1}$ respectively).

Patient 5, who had received 4 weeks of VAPEC-B followed by radiation to the right lower abdomen and pelvis, was azoospermic (pretreatment count of $15 \times 10^6 ml^{-1}$), and patient 14 had a count of $21 \times 10^6 ml^{-1}$ but sperm were non-motile, possibly because of a delay in analysis (semen sample produced at home). Patient 10, who underwent radiotherapy to the right side of pelvis on completion of chemotherapy, had a count of $14 \times 10^6 ml^{-1}$ and was therefore oligospermic ($<20 \times 10^6 ml^{-1}$) by WHO (1980) criteria. This patient was oligospermic before starting treatment (count $15 \times 10^6 ml^{-1}$), but his wife conceived 8 months after the completion of VAPEC-B and has since delivered a healthy infant. Patients 1 and 4, neither of whom had received radiotherapy to sites below the diaphragm, were also oligospermic with counts of 15 and $4 \times 10^6 ml^{-1}$ respectively. Unfortunately, pretreatment semen analysis had not been performed in these patients.

### Discussion

The introduction of MOPP chemotherapy in the early 1960s proved to be a turning point in the treatment of advanced HD (De Vita *et al.*, 1970). Before that time the prospects for long-term survival were poor and concerns over the late effects of cytotoxic drugs on normal tissues were largely irrelevant. However, most patients treated with MOPP and its derivative MVPP (Nicholson *et al.*, 1970) were found to

Correspondence: J.A. Radford.
*Results presented at the Fifth International Conference on Malignant Lymphoma, June 9–12 1993, Lugano, Switzerland.
Received 7 July 1993; and in revised form 29 September 1993.

**Table I** Sperm counts in 14 patients after VAPEC-B chemotherapy for Hodgkin's disease (HD) or non-Hodgkin's lymphoma (NHL)

| *Patient no.* | *Age (years)* | *Diagnosis* | *Weeks VAPEC-B* | *Radiotherapy* | *Months since completion of VAPEC-B* | *Spermatozoa (millions ml$^{-1}$) (pretreatment value)* |
|---|---|---|---|---|---|---|
| 1 | 44 | HD | 4 | Right axilla | 10 | 15 |
| 2 | 36 | HD | 4 | Mantle | 15 | 61 |
| 3 | 31 | HD | 4 | Head and neck | 18 | 215 |
| 4 | 40 | HD | 4 | Head and neck | 5 | 4 |
| 5 | 19 | HD | 4 | Right lower abdomen and right pelvis | 7 | 0 (15) |
| 6 | 31 | HD | 4 | Mantle | 8 | 155 |
| 7 | 25 | HD | 4 | Head and neck | 6 | 123 (250) |
| 8 | 16 | NHL | 11 | Anterior chest wall | 30 | 107 |
| 9 | 45 | NHL | 11 | Left neck | 12 | 95 |
| 10[a] | 24 | NHL | 11 | Right pelvis | 10 | 14 (15) |
| 11 | 28 | NHL | 11 | Mediastinum | 26 | 80 |
| 12 | 37 | NHL | 11 | – | 22 | 209 |
| 13 | 18 | NHL | 11 | Right neck | 20 | 280 (290) |
| 14 | 22 | NHL | 11 | Head and neck | 20 | 21[b] |

[a]This patient's wife conceived 8 months after completing VAPEC-B. [b]Spermatozoa were non-motile.

enter remission and, although 30–40% subsequently relapsed, it was evident that advanced HD was potentially curable using combination chemotherapy. In these circumstances, the long-term effects of treatment on normal tissues are of great importance. This is particularly true of gonadal function in patients with HD, which commonly affects young people who may not have started or completed a family when the diagnosis is made.

Both MOPP and MVPP were found to produce a very high incidence of azoospermia, with only a few men recovering spermatogenesis after treatment (Chapman et al., 1979; Whitehead et al., 1982). Procarbazine, a component of both regimens, causes complete aplasia of the germinal epithelium in the rat (Jackson et al., 1961) and the non-human primate (Sieber et al., 1978), and mustine and vinblastine are also implicated in the germinal epithelial damage following MOPP/MVPP (Spitz, 1948; Vilar, 1975). Of these, only vinblastine is included in the ABVD regimen, which is reported as causing no permanent gonadal damage. Nevertheless, 13 of 24 patients treated with ABVD were found to be azoospermic ($n = 8$) or oligospermic ($n = 5$) a median of 10 months after completion of chemotherapy, and although full recovery of spermatogenesis occurred in all these patients this was not until a further 18 months (median 10 months) had elapsed (Viviani et al., 1985). Furthermore, ABVD is commonly used in combination with MOPP, either as alternating cycles of each regimen (Canellos et al., 1992) or as 'hybridised' cycles of MOPP/ABVD (Viviani et al., 1991), and it seems likely that these treatments cause more severe damage to the germinal epithelium than ABVD alone.

In the present study, only 1 of 14 patients was azoospermic a median of 13.5 months after VAPEC-B. He was oligospermic before starting treatment and had received pelvic irradiation following chemotherapy. A further three men were oligospermic post treatment, and one of these (patient 10) was known to have a subnormal sperm count before starting chemotherapy. All the others ($n = 10$) had counts greater than $20 \times 10^6$ ml$^{-1}$, although spermatozoa from patient 14 were non-motile, possibly because of some delay in analysis.

These results suggest that treatment with VAPEC-B does not cause permanent damage to the male germinal epithelium. On this basis, full assessment of gonadal function is now being performed before and after chemotherapy in a cohort of males and females receiving 11 weeks of VAPEC-B for advanced Hodgkin's disease. However, until the results of this prospective study are available, all male patients will continue to be offered semen cryopreservation before starting treatment.

| *Weeks* | | |
|---|---|---|
| 1 | Doxorubicin 35 mg m$^{-2}$ i.v.<br>Cyclophosphamide 350 mg m$^{-2}$ i.v. | Prednisolone<br>50 mg p.o. daily |
| 2 | Vincristine 1.4 mg m$^{-2}$ i.v.<br>Bleomycin 10 mg m$^{-2}$ i.v. | ↓ |
| 3 | Doxorubicin 35 mg m$^{-2}$ i.v.<br>Etoposide 100 mg m$^{-2}$ p.o. daily for 5 days | |
| 4 | Vincristine 1.4 mg m$^{-2}$ i.v.<br>Bleomycin 10 mg m$^{-2}$ i.v. | |

**Figure 1** The first 4 weeks of VAPEC-B chemotherapy are shown. Subsequent weeks of treatment (to week 11) are repeats of this same basic pattern. In addition to the cytotoxic drugs and prednisolone, prophylactic co-trimoxazole 960 mg twice daily and ketoconazole 200 mg twice daily are prescribed for the duration of treatment.

### References

CANELLOS, G.P., ANDERSON, J.R., PROPERT, K.J., NISSEN, N., COOPER, M.R., HENDERSON, E.S., GREEN, M.R., GOTTLIEB, A. & PETERSON, B.A. (1992). Chemotherapy of advanced Hodgkin's disease with MOPP, ABVD or MOPP alternating with ABVD. *N. Engl. J. Med.*, **327,** 1478–1484.

CHAPMAN, R.M., SUTCLIFFE, S.B., REES, L.H., EDWARDS, C.R.W. & MALPAS, J.S. (1979). Cyclical combination chemotherapy and gonadal function. Retrospective study in males. *Lancet*, **i,** 285–289.

DE VITA, V.T., SERPICK, A.A. & CARBONE, P.P. (1970). Combination chemotherapy in the treatment of advanced Hodgkin's disease. *Ann. Intern. Med.*, **73,** 881–895.

JACKSON, H., FOX, B.W. & CRAIG, A.W. (1961). Anti-fertility substances and their assessment in the male rodent. *J. Reprod. Fertil.*, **2,** 447–465.

NICHOLSON, W.M., BEARD, M.E.J., CROWTHER, D., STANSFELD, A.G., VARTAN, C.P., MALPAS, J.S., HAMILTON FAIRLEY, G. & BODLEY SCOTT, R. (1970). Combination chemotherapy in generalised Hodgkin's disease. *B. Med. J.*, **3**, 7-10.

RADFORD, J.A. & CROWTHER, D. (1991). Treatment of relapsed Hodgkin's disease using a weekly chemotherapy of short duration: results of a pilot study in 20 patients. *Ann. Oncol.*, **2**, 505-509.

RADFORD, J.A., WHELAN, J.S., ROHATINER, A.Z.S., DEAKIN, D.P., HARRIS, M., STANSFELD, A.G., SWINDELL, R., WILKINSON, P.M.W., JAMES, R.D., LISTER, T.A. & CROWTHER, D. (1993). Weekly VAPEC-B chemotherapy for high grade non-Hodgkin's lymphoma; results of treatment in 184 patients. *Ann Oncol.* (in press).

SIEBER, S.M., CORREA, P., DALGARD, D.W. & ADAMSON, R.H. (1978). Carcinogenic and other adverse effects of procarbazine in non human primates. *Cancer Res.*, **38**, 2125-2134.

SPITZ, S. (1948). The histological effects of nitrogen mustard on human tumours and tissues. *Cancer*, **1**, 383-398.

VILAR, O. (1975). Effect of cytostatic drugs on human testicular function. In *Male Fertility and Sterility*, Mancini, R.E. & Martini, L. (eds), Academic Press: New York. pp. 423-440.

VIVIANI, S., SANTORO, A., RAGNI, G., BONFANTE, V., BESTETTI, O. & BONADONNA, G. (1985). Gonadal toxicity after combination chemotherapy for Hodgkin's disease. Comparative results of MOPP versus ABVD. *Eur. J. Cancer Clin. Oncol.*, **21**, 601-605.

VIVIANI, S., BONADONNA, G., SANTORO, A., ZANINI, M., ZUCALI, R., NEGRETTI, E. & VALAGUSSA, P. (1991). Alternating versus hybrid MOPP-ABVD in Hodgkin's disease. The Milan experience. *Ann Oncol.*, **2** (Suppl.), 55-62.

WHITEHEAD, E., SHALET, S.M., BLACKLEDGE, G., TODD, I., CROWTHER, D. & BEARDWELL, C.G. (1982). The effects of Hodgkin's disease and combination chemotherapy on gonadal function in the adult male. *Cancer*, **49**, 418-422.

WORLD HEALTH ORGANIZATION (1980). *Laboratory Manual for the Examination of Human Semen and Semen-Cervical Mucus Interaction*. Belsey, M.A., Moghissi, K.S. Eliasson, R., Paulsen, C.A., Gallegos, A.J. & Prasad, M.R.N. (eds). Press Concern: Singapore. p. 13.